# Global searches for microalgae and aquatic plants that can eliminate radioactive cesium, iodine and strontium from the radio-polluted aquatic environment: a bioremediation strategy

**DOI:** 10.1007/s10265-013-0596-9

**Published:** 2013-12-18

**Authors:** Shin-ya Fukuda, Koji Iwamoto, Mika Atsumi, Akiko Yokoyama, Takeshi Nakayama, Ken-ichiro Ishida, Isao Inouye, Yoshihiro Shiraiwa

**Affiliations:** Faculty of Life and Environmental Sciences, University of Tsukuba, 1-1-1 Tennodai, Tsukuba, Ibaraki 305-8572 Japan

**Keywords:** Algal phytoremediation, Bioaccumulation, Radiopollution, Radionuclide elimination, Radioactive cesium, The Fukushima 1 Nuclear Power Plant accident

## Abstract

The Fukushima 1 Nuclear Power Plant accident in March 2011 released an enormously high level of radionuclides into the environment, a total estimation of 6.3 × 10^17^ Bq represented by mainly radioactive Cs, Sr, and I. Because these radionuclides are biophilic, an urgent risk has arisen due to biological intake and subsequent food web contamination in the ecosystem. Thus, urgent elimination of radionuclides from the environment is necessary to prevent substantial radiopollution of organisms. In this study, we selected microalgae and aquatic plants that can efficiently eliminate these radionuclides from the environment. The ability of aquatic plants and algae was assessed by determining the elimination rate of radioactive Cs, Sr and I from culture medium and the accumulation capacity of radionuclides into single cells or whole bodies. Among 188 strains examined from microalgae, aquatic plants and unidentified algal species, we identified six, three and eight strains that can accumulate high levels of radioactive Cs, Sr and I from the medium, respectively. Notably, a novel eustigmatophycean unicellular algal strain, nak 9, showed the highest ability to eliminate radioactive Cs from the medium by cellular accumulation. Our results provide an important strategy for decreasing radiopollution in Fukushima area.

**Electronic supplementary material:**

The online version of this article (doi:10.1007/s10265-013-0596-9) contains supplementary material, which is available to authorized users.

## Introduction

The F1NPP accident led to the discharge of a large quantity of radioactivity into the environment. The gross amount released from the power plant was estimated to be 900 PBq (TEPCO 2012) and the radionuclides were distributed widely into 30 km area around the power plant (Chino et al. 2011). Radioactive Cs, Sr and I released into the air were estimated at 10–37 PBq, 150 TBq and 90–500 PBq, respectively (Chino et al. 2011; Nuclear Emergency Response Headquarters 2011; Stohl et al. 2012; TEPCO 2012). Among them, radionuclides released into the ocean were reported as 940 TBq ^134^Cs, 940 TBq ^137^Cs, 90–900 TBq ^90^Sr, and 2.8 PBq ^131^I (Casacuberta et al. 2013; Nuclear Emergency Response Headquarters 2011). In addition, TEPCO has been injecting a large volume of water into the F1NPP reactors for cooling, and a total volume of ~93,370 m^3^ of highly polluted water had been stored in the reactor and extra tanks by 27 March 2013 (TEPCO 2013). Unfortunately, the amount of radio-polluted water is further increasing day-by-day due to the continuous operation of cool water injection and the incurrent of underground water into the defective reactor. Thus, it is our urgent task to safely recover such a large volume of highly radio-polluted water and eliminate radionuclides below environmentally safety levels.

Among the radionuclides released into the environment, ^134^Cs, ^137^Cs, ^90^Sr, and ^129^I are easily taken up by organisms, since Cs and Sr are analogs of potassium and calcium, respectively, and iodine is an essential element for many organisms. On the other hand, ^89^Sr, ^131^I, ^132^I, ^133^I and ^135^I, which were also released into the environment, decay rapidly due to their short half-life times, i.e., 50.5, 8, 2.3, 0.88 and 0.27 days for ^89^Sr, ^131^I, ^132^I, ^133^I and ^135^I, respectively. In addition, ^129^I was not released directly but has been produced secondarily by the disintegration of released ^129m^Te (NISA 2011).

The artificial radionuclides ^137^Cs and ^90^Sr are monitored in total diet studies in terms of risk management in many countries. ^90^Sr is of particular concern because of its long half-life (28 years) and its potential risk of deposition in bone (Betsy et al. 2012). On the other hand, iodine is an essential element for higher animals but has negative effects at high concentrations. Some organisms such as brown macrophytes accumulate high concentrations of iodine in the algal body (Carolan et al. 2011; Chowdhury and Blust 2011; Gall et al. 2004; Iwamoto and Shiraiwa 2012; Küpper et al. 1998; Martinelango et al. 2006; Marzano et al. 2000; Rowan and Rasmussen 1994). When organisms ingest drinking water or foods with heavy radiopollution or breathe polluted air, it would increase the risk of suffering radiation problems (Escher and Hermens 2004; Golikov et al. 2004). Because radionuclides can be highly concentrated within the food web, the problems become bigger with time (Dubrova et al. 1996; Fugazzola et al. 1995; Nikiforov and Gnepp 1994). Therefore, a removal of radionuclides from the environment is an urgent task to reduce the risk brought by the radiopollutants.

The development of new technology and engineering strategies are critical to decontamination of radionuclides, which are distributed widely in both terrestrial and aquatic environments at very low concentrations. Chemical methods such as precipitation and adsorption seem not effective for such low quantity radionuclides. Therefore, biological processes or bioremediation strategies are potentially important.

Many terrestrial bioremediation methods have employed terrestrial plants to eliminate radionuclides from the environment since the Chernobyl accident in 1986 (IAEA 2006). The accumulation of radioactive Cs into terrestrial plants such as tea (Mück 1997; Polar 2002), rice (Hosono and Takahashi 2013; Nakanishi et al. 2013; Tanoi et al. 2013), sunflower (Dushenkov et al. 1997), and tomato (Endo et al. 2013) have been reported. In other studies of this issue, the effects of radionuclides released from F1NPP on terrestrial plants have been discussed (Kawai et al. 2014; Kobayashi et al. 2014; Mimura et al. 2014; Ohmori et al. 2014; Sekimoto et al. 2014; Terashima et al. 2014; Yamashita et al. 2014).

In this study, we investigated the ability of algae and aquatic plants to eliminate radionuclides from media, in order to establish a strategy for decontaminating the aquatic environment in Fukushima area. We have screened 188 strains of algae and aquatic plants for strains that possess the ability to efficiently accumulate radionuclides and, hence, are potentially useful for eliminating the radionuclides from the environment.

## Materials and methods

### Algae and aquatic plants and their culture conditions

**Fig. 1:**
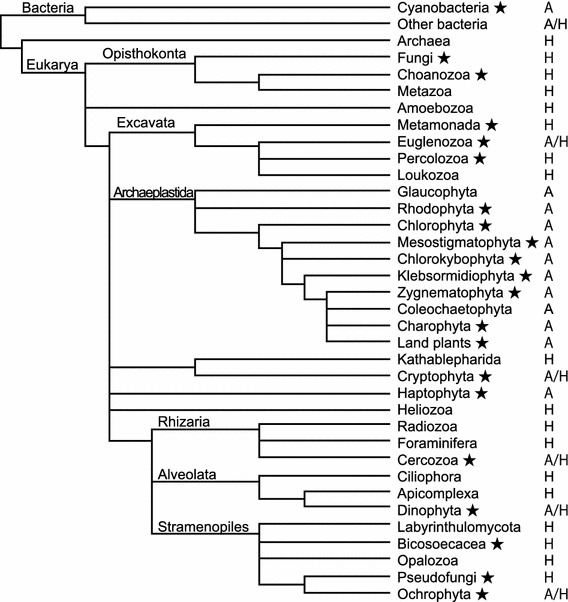
Phylogenic position of experimental organisms in the schematic phylogenetic tree of life (phylum-level). *Star*, phylum including strains examined in this study; *A*, *H* and *A/H*, nutrient condition indicating mostly autotrophic, mostly heterotrophic and mixture of *A* and *H*, respectively

In total, 188 strains of algae and aquatic plants were used in this study. Of these strains, 99 strains were from the culture collection of our group; 75 strains coded with NIES were from the culture collection of the National Institute for Environmental Studies (NIES) in Tsukuba, Japan; five strains Is-ta-kw, Ch-deb-2, Ch-n-kw, Th-n-5, and Nd23 × Nd36-3 were provided by Dr. Akira Kuwata (Tohoku National Fisheries Research Institute); five strains coded as TIR 1 to TIR 5 were provided by Ms. Misaka Taira (University of Tsukuba); and four strains coded as We 1 to We 4 were purchased from a local pet shop in Tsukuba, Japan (see Table S1). The strains used in this study were phylogenetically broad organisms classified in cyanobacteria and eukaryotes such as Opistokonta, Excavata, Archaeplastida, Rhizaria, Alveolata and Stramenopiles, as shown in Fig. 1. These organisms show various features in their morphology, physiology and biochemical properties. They also show various nutritional properties such as autotrophic and/or heterotrophic and seawater tolerant or intolerant.

For stock and pre-experimental cultures, marine algal strains were grown in seawater enriched either with ESM, IMK or f/2 medium (Kasai et al. 2004), and freshwater algae and aquatic plants were grown either in C, CS, or AF-6 medium (Kasai et al. 2004).

For radionuclide elimination testing, each freshwater medium was prepared without potassium. Heterotrophs were grown in AF-6 or seawater medium containing organic nutrients such as GPY (4 g l^−1^ glucose, 2 g l^−1^ polypeptone, and 1 g l^−1^ yeast extract) or YT (2 g l^−1^ triptone and 1 g l^−1^ yeast extract) (Table S1). All strains were incubated at 20 °C under continuous light of 100 μmol photon m^−2^ s^−1^ for several days as indicated in the text.

### Global searches for strains with high radionuclide elimination ability

One hundred and eighty-eight strains were grown and tested for their ability to eliminate radionuclides from the medium (Table S1). The test was initiated by the addition of 1,000 Bq ml^−1^ radionuclides into cultures containing individual strains suspended in 15 ml fresh medium. In our global searches the elimination ability of radionuclides was primarily assessed because this ability is important in our strategy to remove radionuclides from the environment using aquatic plants and algae. After starting cultures, algae and aquatic plants grow differently and therefore the biological mass of individual cultures and strains differed at similar time points. Thus, for normalization, the values of radionuclide elimination ability factored in both the growth activity and the absorption/adsorption activity of radionuclides by organisms. Radionuclides used in this study were 3.7 MBq ml^−1^ ^137^CsCl (Specific activity, 61.7 GBq mmol l^−1^; Eckert and Ziegler Isotope Products, Valencia, CA, USA) and 217.2 MBq ml^−1^ ^85^SrCl_2_ (Specific activity, 17.6 GBq mmol l^−1^; Perkin Elmer, Inc., Waltham, USA) and 3.7 G Bq ml^−1^ ^125^I (carrier free; MP Biomedicals, Inc., Santa Ana, USA). ^125^I was added as a mixture of iodide (I^−^) and iodate (IO_3_^−^) in a 1:1 ratio. Iodide was chemically oxidized to iodate by adding 2.0 % H_2_O_2_ to the stock solution (Liebhafsky et al. 1978). The concentrations of radionuclides were 2.2 and 7.1 ng ml^−1^ for Cs and Sr, respectively. However, the value of iodine-radionuclide could not be calculated exactly since carrier-free ^125^I was added to seawater containing 5.9 μg ml^−1^ I.

In our global searches, disposable plastic flasks were used as reaction vessels (Culture Flask, 3100-025, IWAKI, Tokyo, Japan). The radionuclides were added to the reaction medium containing algae or aquatic plants previously grown for 24 h. After injection of radionuclides, an aliquot of culture (100 μl) was taken out at 0, 7 and 14 days for photoautotrophs, and 0, 4 and 7 days for heterotrophs. For microalgae, samples were separated into the medium and cell fractions by a silicone-oil layer centrifugation method, as described in our previous paper (Araie et al. 2011).

In case of aquatic plants, each culture medium was also subjected to the same silicone-oil layer centrifugation method to separate small particles from the media. Radioactivity was determined using a gamma-ray counter (Aloka Accuflex γ7000, Tokyo, Japan). The elimination ability of radionuclides was calculated as the difference in the radioactivity in the medium at time 0 and at each sampling time. All experiments were performed at least twice to confirm repeatability of our tests, using multiple cultures for each strain.

### Radionuclide elimination tests for selected strains

In our global searches, strains that showed an ability to efficiently eliminate radionuclides from the medium (more than 40 %) were selected and subjected to the secondary test to increase the reliability of our data. Radioactivity measurements were carried out for samples at 0, 2, 4 and 8 days after the addition of each radionuclide. The same experiments were repeated three times for selected strains. In each test, the sample biomass was determined by growing one mock (control) culture that contained no radionuclide: at 8 days of culture, control cells were harvested by centrifugation or filtration, washed with distilled water, and then dried by freeze-drying for ca. 12 h using Lyph Lock 6 (Labconco, Kansas City, MO, USA) according to the manufacturer’s instruction. Dried samples were weighed on an electric precision balance (AG285, Mettler-Toledo, Greifensee, Switzerland).

The radioactivity partitioned in the cells, the cell-free medium, and the residual precipitates including particles attached onto a culture flask wall was measured. After 8 days of incubation with radionuclides, cells and medium were separated by passing through a glass filter (Whatman GF/F, GE Healthcare, Buckinghamshire, UK). The cells on the filter and the vessels were rinsed with 15 ml each of distilled water twice. Filtrates and medium were combined and stored as a medium fraction. Radionuclides adsorbed on the culture flask wall were eliminated by solubilizing with 5 ml of 5 % sodium dodecyl sulfate and stored as an adherence fraction. The total radioactivity accumulated in filtered cells was measured together with the filter.

## Results

### Global searches for strains with high radionuclide elimination ability

**Fig. 2:**
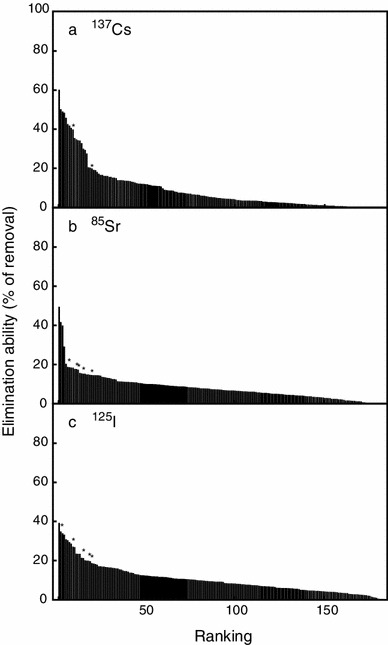
Elimination ability of radionuclides from the culture medium by algae and aquatic plants in the primary (global) screening. **a** ^137^Cs; **b** ^85^Sr; **c** ^125^I. Average values are ranked in descending order. List of all organisms and values are the same as those listed in Table S1. Seawater strains ranked within top 20 in each radionuclide are marked by *asterisks*

In order to select algae and aquatic plants that can be served for eliminating radionuclides from the aquatic environment, we searched for stains that accumulated high levels of radioactive Cs^+^, Sr^2+^ and I^−^ from the medium as described in the “Materials and methods”. Among 188 strains of algae and aquatic plants examined, ^137^Cs, ^85^Sr and ^125^I were significantly eliminated by 167, 181 and 187 strains, respectively. These organisms are shown in descending order of average ability to eliminate radionuclide from the medium (Fig. 2).

### Evaluation of radionuclide elimination ability for selected strains

**Fig. 3:**
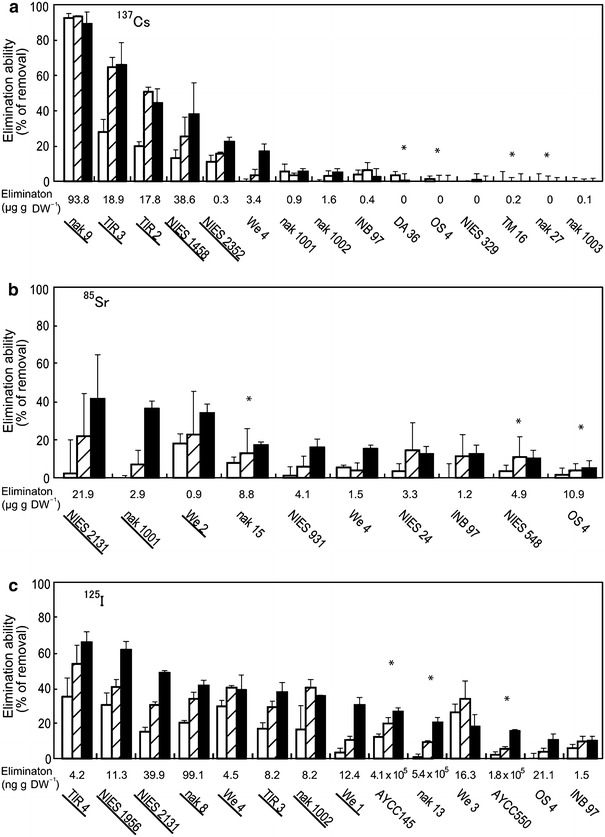
Elimination ability of radionuclides from the culture medium by selected algae and aquatic plants in the second screening. **a** ^137^Cs; **b** ^85^Sr; **c** ^125^I. *Opened*, *hatched* and *closed bars* represent values of percent elimination after 2-, 4- and 8-days incubation, respectively. The values (ng g DW^−1^) indicate the mass of radionuclides accumulated/recovered by cells during 8 days (calculated on the basis of dry weight). The names and strain codes (marked with *underline*) are the same as those listed in Table 1. Others are as followings: AYCC145, *Bangia* sp., Rhodophyta; AYCC550, *Bangiopsis* sp., Rhodophyta; DA 36, *Heterosigma akashiwo*, Ochrophyta; INB 97, *Coelastrum* sp., Chlorophyta; nak 13, *Tetraselmis* sp., Chlorophyta; nak 15, *Tetraselmis* sp., Chlorophyta; nak 27, *Calyptrosphaera sphaeroidea*, Haptophyta; nak 1003, *Spirogyra* sp., Zygnematophyta; NIES-329, *Ulothrix variabilis*, Chlorophyta; NIES-548, *Acinetospora crinite*, Ochrophyta; NIES-931, *Gloeocapsa decorticans*, Cyanobacteria; OS 4, *Dixoniella grisea*, Rhodophyta; TM 16, *Amphidinium massartii*, Dinophyta; and We 3, *Eleocharis acicularis*, Tracheophyta (See also Table S1). Strains We 4 and OS 4 were additionally tested in this screening. Seawater strains are indicated by *asterisks*

Based on the primary data in Fig. 2, 15, 10 and 14 strains with significantly high elimination ability for ^137^Cs, ^85^Sr and ^125^I, respectively, were selected for further tests (Fig. 3). The elimination time courses of the three radionuclides suggested that radionuclides were mostly eliminated by absorption, but not by simple adsorption onto the surface, since the values increased slowly and rather linearly with incubation times.

Five strains were selected as highly positive, radioactive Cs eliminators (Table 1). They include three microalgae such as an unidentified freshwater eustigmatophycean strain, nak 9 (ca. 90 % elimination), the freshwater floridephycean *Batrachospermum virgato*-*decaisneanum* NIES-1458 (ca. 38 % elimination), the chlorophyte *Chloroidium Saccharophilum* NIES 2352 (ca. 22 % elimination) and the two aquatic plants (trachiophytes) *Lemna aoukikusa* TIR 2 and TIR 3 (ca. 45 and 66 % elimination, respectively) (Table 1). Notably, nak 9 displayed an exceptional ability of swift elimination, i.e., as fast as 2 days to reach the steady state level (Fig. 3a).

**Table 1:** List of strains which showed high radionuclide elimination abilities from the medium in assay for selected strains

RI	Phylum	Species	Strain code	Elimination ability (%)			Medium	Habitat
				^137^Cs	^85^Sr	^125^I		
^137^Cs	Eustigmatophyceae	Unidentified	nak 9	89.2	–	–	AF6	Freshwater
	Tracheophyta	*Lemna aoukikusa*	TIR 3*	66.0	–	37.6	C	Freshwater
	Tracheophyta	*Lemna aoukikusa*	TIR 2*	44.5	–	–	C	Freshwater
	Florideophyceae	*Batrachospermum* *virgato*-*decaisneanum*	NIES-1458	37.9	–	–	C	Freshwater
	Chlorophyta	*Chloroidium* *saccharophilum*	NIES-2352	22.4	–	–	AF6	Freshwater
^85^Sr	Cyanophyceae	*Stigonema ocellatum*	NIES-2131	–	41.3	48.5	AF6	Freshwater
	Chlorophyceae	*Oedogonium* sp.	nak 1001*	5.6	36.3	–	AF6	Freshwater
	Magnoliopsida	*Egeria densa*	We2*	–	33.9	–	AF6	Freshwater
^125^I	Cyanophyceae	*Nostoc commune*	TIR 4*	–	–	65.9	C	Terrestrial
	Cyanophyceae	*Scytonema javanicum*	NIES-1956	–	–	61.9	C	Terrestrial
	Cyanophyceae	*Stigonema ocellatum*	NIES-2131	–	41.3	48.5	C	Freshwater
	Xanthophyceae	*Ophiocytium* sp.	nak 8	–	–	41.6	AF6	Freshwater
	Chlorophyta	*Elodea nuttallii*	We 4	17.1	15.4	38.8	AF6	Freshwater
	Tracheophyta	*Lemna aoukikusa*	TIR 3*	66.0	–	37.7	C	Freshwater
	Chlorophyta	*Rhizochlonium* sp.	nak 1002	5.1	–	35.6	AF6	Freshwater
	Magnoliopsida	*Cabomba caroliana*	We 1*	–	–	30.6	C	Freshwater

The values of elimination ability are average in three multiple independent experiments. Strains were incubated for 2, 4 and 8 days. Medium: culture media. Habitat: original habitat where each strain was isolated. *: not bacteria-free

Three strains showed a high ability to eliminate radioactive Sr. They were the cyanobacterium *Stigonema ocellatum* NIES-2131 (ca. 41 % elimination), the chlorophycean alga *Oedogonium* sp. nak 1001 (ca. 36 % elimination) and the Magnoliopsidae *Egeria densa* We2 (ca. 34 % elimination) (Table 1).

For radioactive I elimination, five stains were promising. They were the cyanophyceans *Nostoc commune* TIR 4 (ca. 66 % elimination), which shows very high drought tolerance (Fukuda et al. 2008), *Scytonema javanicum* NIES-1956 (ca. 62 % elimination) and *Stigonema ocellatum* NIES-2131 (ca. 49 % elimination), the freshwater xanthophycean alga *Ophiocytium* sp. nak 8 (ca. 42 % elimination), and the aquatic vascular plant *Elodea nuttallii* We1 (ca. 31 % elimination) (Table 1).

**Fig. 4:**
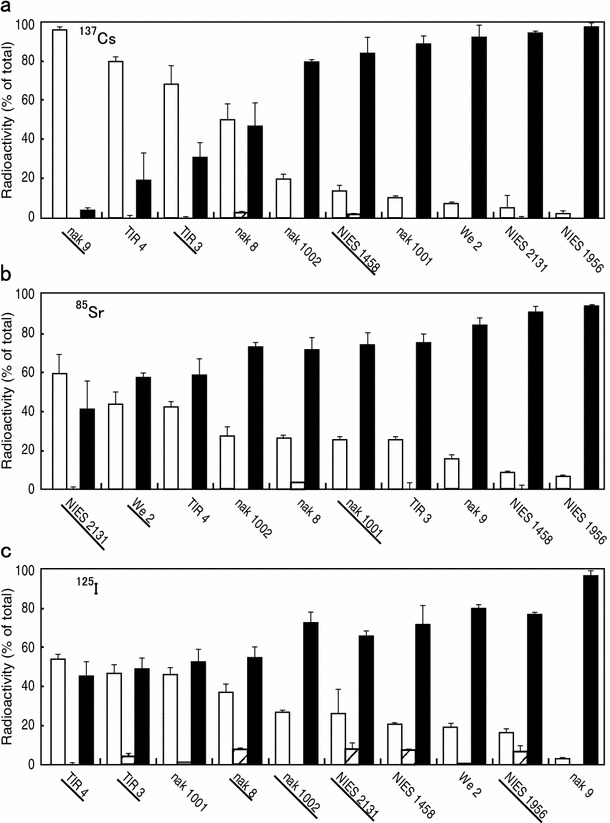
Fractionation of radionuclides from the strain culture medium. After incubation with ^137^Cs (**a**), ^85^Sr (**b**) and ^125^I (**c**) for 8 days, the radioactivity recovered in the cells (*open bar*), the precipitates (*hatched bar*) and the soluble materials (*closed bar*) from the medium was determined. The names of strains are the same as shown in Fig. 3, except nak 8 (*Ophiocytium* sp., Ochrophyta) and TIR 4 (*Nostoc commune*, Cyanobacteria) (See also Table S1). All these strain codes are *underlined* in Table 1

Where did the organisms accumulate the radionuclides absorbed from the medium? To examine this, cells and organisms were incubated with radionuclides for 8 days and then disrupted to separate three fractions, i.e., the cells, the cell-free culture medium, and insoluble precipitates including precipitates from the medium and those recovered from the culture flask wall. Figure 4 clearly shows that radionuclides were mainly deposited into either cells or the cell-free culture medium, but very little into the insoluble materials.

### Selection of useful strains with an ability to eliminate multiple radionuclides

**Fig. 5:**
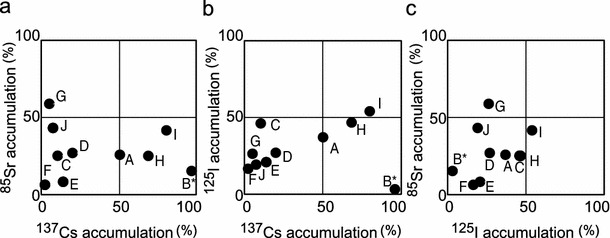
Comparison between the abilities of selected strains to eliminate two different radionuclides in the radionuclide elimination test. **a** ^137^Cs vs. ^85^Sr; **b** ^137^Cs vs. ^125^I; **c** ^125^I vs. ^85^Sr. Strains: *A* nak 8; *B* nak 9; *C* nak 1001; *D* nak 1002; *E* NIES-1458; *F* NIES-1956; *G* NIES-2131; *H* TIR 3; *I* TIR 4; *J* We 2; and *asterisk* unicellular strain. The names of strains are listed in Figs. 3 and 4 (See also Table S1)

Some of microalgae and aquatic plants eliminated multiple radionuclides from the medium (Fig. 5). *L. aoukikusa* (Tracheophyta) TIR 3 and TIR 4 exhibited relatively high elimination efficiency (25–78 % elimination) for ^137^Cs, ^85^Sr and ^125^I from the media (Fig. 5, marked with *H* and I). However, we could not find any strain that could efficiently eliminate all of the above radionuclides from the medium (>90 % elimination).

### Effect of potassium on radioactive cesium elimination

**Fig. 6:**
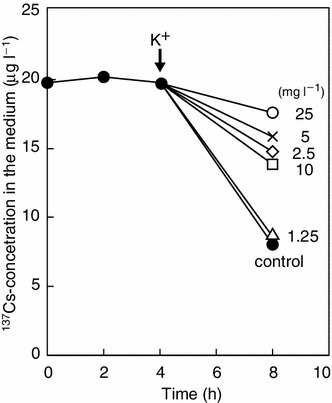
Effect of potassium on the ability of the eustigmatophycean strain nak 9 to eliminate radioactive Cs. Algal cells were firstly grown in the potassium-deficient AF-6 medium for 14 days and then transferred to the fresh medium containing ^137^Cs (10 MBq l^−1^). Various concentrations of potassium phosphate were added at 4 h, as indicated by an arrow. Elimination of ^137^Cs from the medium was determined by the silicone-oil-layer-centrifugation method, as described in “Materials and methods”. Final concentration of potassium (mg l^−1^): *Closed circle* 0 (control); *triangle* 1.25; *diamond* 2.5; *×-mark* 5; *square* 10; *open circle* 25

The freshwater unidentified eustigmatophycean strain nak 9 was the best microalgae for radioactive Cs accumulation/elimination (Table 1; Figs. 3, 4). Using this strain, the effect of potassium on Cs-elimination ability was tested (Fig. 6). The elimination ability became substantial after 4 h incubation, but this ability was suppressed by exogenously supplied potassium, depending on the concentration of potassium. These results suggested that ^137^Cs is taken up by the cells in a competitive manner with potassium (Fig. 6). However, the exact mechanism for ^137^Cs absorption by the cells remains to be elucidated (see “Discussion”).

## Discussion

### Global searches for strains with promising radionuclide elimination ability

We tested the ability of cells to accumulate/eliminate ^137^Cs, ^85^Sr and ^125^I from the medium among 188 strains of microalgae, aquatic plants, and colorless protists (Table S1; Fig. 2). We showed that algae and aquatic plants gradually absorb these radionuclides in a day-order time course (Fig. 3). We also showed that fresh water strains exhibited high ability for eliminating not only ^137^Cs, but also ^85^Sr and ^125^I (Table 1). Among the terrestrial algal strains, the multicellular filamentous cyanobacteria *Nostoc commune* TIR 4 and *Scytonema javanicum* NIES-1956 exhibited an especially high ability of I-elimination. However, we could find no marine strains which exhibit highly efficient elimination ability for the radionuclides in this study. This may be due to the presence of competitive elements in the seawater, such as potassium, calcium and non-radioactive iodine, which should decrease the efficiency of either absorption or accumulation of radionuclides by the cells. Colonial or multicellular strains had a tendency to eliminate ^125^I efficiently (Table 1, Table S1), although the mechanism remains unclear.

Heterotrophic organisms also showed lower uptake ability compared with autotrophic organisms (Fig. 3). This may also be related to the presence of physiological levels of potassium and calcium in the yeast extracts, which are supplemented to the growth medium for heterotrophs (Table S1).

When compared among various taxa, cyanobacteria, green algae and ochrophytes seem to exhibit higher radionuclide uptake ability than organisms in other taxa. This difference may be due to the presence of cell walls in these algal groups. However, rhodophytes, which also have a thick cell wall and mucoid substances, exhibited low radionuclide elimination ability, suggesting that the cell walls play a negative role against radionuclide uptake by the rhodophytes.

In our second tests for selected strains, five, three and eight strains showed high ability for eliminating ^137^Cs, ^85^Sr and ^125^I, respectively, from the medium (Table 1). Because these plants are easy to harvest and dry, they must be potentially useful to recover radioactive Cs from a huge volume of radio-polluted water.

### Elimination of radioactive cesium

In our global searches, highly active strains for eliminating radioactive Cs, Sr and I are all fresh water strains. Cs, an alkali metal, is particularly known to be transported into cells as an analog of potassium. Therefore, the presence of potassium in the medium strongly disturbs the uptake of Cs (Bystrzejewska-Piotrowska and Urban 2003; Cline and Hungate 1960; Plato and Denovan 1974; Shaw 1993). For example, Cs uptake ability was reported to decrease by more than 80 % when 1.3 mM potassium was present in the medium with IC_50_ value of ca. 0.6 mM potassium (Plato and Denovan 1974). Such inhibition by potassium is confirmed in the present study. Radioactive Cs accumulation by the eustigmatophycean strain nak 9 was suppressed by potassium depending on the potassium concentration (Fig. 6). In fresh water, potassium concentrations are generally 0.5–3 mg l^−1^ and therefore the inhibition of Cs uptake by potassium should not be observed in the field. In contrast, seawater contains 390 mg l^−1^ potassium (Turekian 1968), and our results are consistent with a view that marine algae are unlikely to show an effective elimination ability for radioactive Cs.

In higher plants, sunflower has been reported to absorb 150 μg Cs in 100 h (Dushenkov et al. 1997), whereas a vetiver (*Vetiveria zizanoides*) absorbed 61 % of ^137^Cs in 168 h from radio-polluted water produced in the Chernobyl accident (Singh et al. 2009). In algae, the brown alga *Laminaria digitata* adsorbed more than 80 % of ^134^Cs under high pH conditions when the membrane was phosphorylated artificially (Pohl and Schimmack 2006). In this study, we found that the eustigmatophycean alga nak 9 could eliminate more than 90 % of radioactive Cs without any special treatment (Figs. 3, 4). The removal rate of 46.9 mg Cs kg DW^−1^ day^−1^ was very high (Figs. 3, 6). Therefore, we suspect that Cs is adsorbed on to the cell surface, although further study is needed to determine whether radioactive Cs is adsorbed by extra-cellular materials or absorbed into cells through the membrane (Figs. 3, 6).

### Elimination of radioactive strontium

Strontium is known to accumulate in organisms and behave as an analog of calcium (Comar et al. 1957). In general, the elimination ability for radionuclides is believed to partly depend on the amount of gelatinous polysaccharide materials covering cell surface (Hill et al. 1997; Tamaru et al. 2005). Such extracellular polysaccharides have been reported to adsorb heavy metals. In the cyanobacterium *Synechocystis* sp. PCC6803, an extracellular hemolysin-like protein (HLP) conjugate with polysaccharides functions to adsorb ^109^Cd very rapidly (in a minute-order time course) (Sakiyama et al. 2011). In *N. commune*, the absorption of ^85^Sr into the cells is increased by phosphorylation on the cell surface (Pohl and Schimmack 2006). In terrestrial plants, sunflower is able to entirely absorb 150 μg Sr in 100 h when grown in radio-polluted water (Dushenkov et al. 1997). On the other hand, our results with algae and aquatic plants showed linier increase in elimination ability during time course (Fig. 3). These data suggest that ^85^Sr can be eliminated from the medium by absorption, but not by simple adsorption.

### Elimination of radioactive iodine

We showed that the uptake ability of ^125^I is high in cyanobacteria, green algae (especially Chlorophyceae, Ulvophyceae, and streptophyte algae) and ochrophytes. The top three eliminators of ^125^I were cyanobacteria (Table 1; Figs. 2, 3). It is not yet well understood why many living organisms require iodine, although brown macroalgae contain high amounts of iodine in the fronds (Gall et al. 2004; Phaneuf et al. 1999). In some microalgae, iodine is known to accumulate in the cells, but the effects are diverse, i.e., it stimulates, suppresses or exerts no effect on the growth of marine microalgae depending on species (Iwamoto and Shiraiwa 2012). Iodine is absorbed by microalgal cells as iodide (I^−^) and iodate (IO_3_^−^), the latter being predominant in seawater (Iwamoto and Shiraiwa 2012). In terrestrial plants, iodide is highly concentrated in the roots of Japanese mustard spinach (Muramatsu et al. 1983), and paddy-rice accumulates considerable amounts of ^131^I adsorbed from soil in the roots (Tensho and Yeh 1970).

### Effect of potassium on radioactive cesium elimination by the strain nak 9

Figure 5 shows that *Lemna aoukikusa* (TIR 3), a floating vascular plant, is useful for the elimination of both radioactive Cs and I and that the cyanobacterium *Stigonema ocellatum* (NIES-2131) is useful for the removal of both Sr and I. Besides of these organisms, elimination ability of radioactive Cs, Sr and I is species-specific for each element. Radioactive Sr can be eliminated selectively by the chlorophycean alga *Oedogonium* sp. (nak 1001) and radioactive I can be eliminated by a chlorophycean vascular aquatic plant *Elodea nuttallii* (We 4) and an ulvophycean filamentous alga *Rizochlonium* sp. (nak 1002).

### Future study

According to the TEPCO report, water polluted with radioactive Cs is continuously stored in the main facilities of F1NPP such as the nuclear reactor building and turbine building (TEPCO 2013). Reducing the amount of radio-polluted soils and water is an urgent task in our society. Therefore, biological concentration of radionuclides is an essential technology for bioremediation of the radio-polluted environment. In this study, we succeeded in identifying some microalgae, such as the eustigmatophycean strain nak 9, which are potentially useful for decontaminating radioactive Cs from highly radio-polluted water stored in the nuclear reactor building of F1NPP, or for reducing a volume of the radio-polluted water. However, in practical application of our strains to decontamination of mega volumes of radio-polluted water in F1NPP, further studies are required to develop a system that allows mass cultivation and efficient coagulation/sedimentation of algal strains. It is also important to find new strains that possess high ability to eliminate multiple radionuclides i.e. Cs, Sr and I from the medium.

It is also important to develop a method for solubilizing Cs from soil matrix. In the environment, such as river water, ponds and the sea surrounding the radiopolluted area, the radioactivity of radiopollutants released from the F1NPP was below a detectable limit (FA 2013). Furthermore, radioactive Cs has been reported to tightly bind to fine soil particles in shallow depth. Radioactive Cs is extremely difficult to extract from the sediment particles and needs to be treated with extremely strong acid for re-solubilizing. In order to use microalgae for eliminating radioactive Cs from the soil, we must first need to release Cs from the fine soil particles into aqueous solution. As one of possible methods, an electrokinetic method reported recently (Oguri et al. 2004) would be suitable for releasing such tightly bound Cs into water. In combination with such treatment, the usefulness of our microalgal phytoremediation must be enhanced by further studies on technological development under tight collaboration of scientists among different scientific disciplines.

## Electronic supplementary material

Below is the link to the electronic supplementary material.

Supplementary material 1 (DOCX 36 kb)
