# Supplementary material for: Global searches for microalgae and aquatic plants that can eliminate radioactive cesium, iodine and strontium from the radio-polluted aquatic environment: a bioremediation strategy
Source: J Plant Res. 2013 Dec 18;127(1):79–89. doi: 10.1007/s10265-013-0596-9 (PMC3889918; doi:10.1007/s10265-013-0596-9)
Supplement: Supplementary file 1 — Supplementary material 1 (DOCX 36 kb) [file 10265_2013_596_MOESM1_ESM.docx]

**Table S1** List of strains and their radionuclide elimination abilities examined for global screening. Elimination ability was calculated as an average value using the maximum value in multiple independent experiments. Autotrophs and heterotrophs were incubated for 7 and 14 day, and 4 and 7 day, respectively. The maximum values are cited. Medium: culture media. Habitat: original habitat where each strain was isolated.*, not axenic;**, containing 10% seawater;***,containing 50% seawater; BW: brackish water;＋YT, including yeast extracts (1 g L^-1^) and tryptone(2 g L^-1^);＋GPY, including glucose(1 g L^-1^), polypeptone(1 g L^-1^) and yeast extracts (1 g L^-1^). Media with +YT and +GPY are heterotrophic.

Supergroup Phylumand Class Species Strain code Elimination ability (%) Medium Habitat

^137^Cs ^125^I ^85^Sr

Bacteria Cyanobacteria *Acaryochloris marina* NIES-2412 0 21.8 5.9 IMK Sea water

Cyanophyceae *Aphanizomenonflos-aquae* NIES-1728 12.5 14.4 20.3 C Freshwater

*Arthrospiraplatensis* NIES-597 32.5 32.5 9.5 C Freshwater

*Chroogloeocystissiderophila* NIES-1031 1.6 32.3 12.6 C Hot spring

*Cylindrospermopsisraciborskii* NIES-1259 26.3 26.3 16.0 C Freshwater

*Gloeocapsadecorticans* NIES-931 0 17.1 45.6 C Terrestrial

*Microcystisaeruginosa* NIES-843 9.7 19.7 4.3 C Freshwater

*Nostoc commune* TIR 4* 2.9 45.2 1.6 C Terrestrial

*Nostoc commune* NIES-24 0.8 15.8 92.4 C Terrestrial

*Oscillatorialimnetica* NIES-36 3.9 6.6 20.0 C Terrestrial

*Phormidiumfoveolarum* NIES-503 2.2 13.8 18.2 C Freshwater

*Scytonemajavanicum* NIES-1956 6.2 60.1 7.2 C Terrestrial

*Stigonemaocellatum* NIES-2131 10.6 45.0 79.2 AF6 Freshwater

Opisthokonta Ascomycota Unidentified CR 54-1 8.0 8.0 13.8 AF6+GPY Freshwater

Unidentified CR 49-4 11.9 14.8 9.8 AF6+GPY Freshwater

Unidentified CR 48-4 5.4 5.4 11.7 AF6+GPY Freshwater

Unidentified CR 14-6 13.1 13.1 19.3 IMK+GPY Sea water

Unidentified CR 14-4 6.5 11.7 10.4 IMK+GPY Sea water

Unidentified CR 13-7 0 12.6 3.3 IMK+GPY Sea water

Unidentified CR 13-2 3.0 8.2 13.3 IMK+GPY Sea water

Unidentified CR 12-7 2.9 2.9 15.8 AF6+GPY Freshwater

Unidentified CR 12-1 2.6 7.4 14.9 AF6+GPY Freshwater

Unidentified CR 9-9 11.9 11.6 11.6 IMK+GPY Sea water

Choanozoa

Choanoflagellatea *Salpingoecainfusionum* NIES-1442 0 9.2 7.5 IMK+YT Sea water

Excavata Euglenozoa

Kinetoplastea *Bodosaltans* NIES-1439 4.4 5.7 10.5 AF6+YT Freshwater

Euglenophyceae *Euglena* sp. INB 80 8.5 8.9 13.0 AF6 Freshwater

*Euglena* sp. INB 92 6.9 13.9 0.8 AF6 Freshwater

*Euglenaria anabaena* INB 108 2.9 11.3 0 AF6 Freshwater

*Eutreptiella* sp. NIES-2325 10.6 12.0 7.9 IMK Sea water

*Strombomonas* sp. INB 1 0.8 25.5 26.2 AF6 Freshwater

*Trachelomonas* sp. INB 57 7.3 16.6 11.7 AF6 Freshwater

Metamonada

Trepomonadea *Hexamita* sp. NIES-1440 7.5 7.5 10.7 AF6+YT Freshwater

Percolozoa

Heterolobosea *Percolomonas* sp. NIES-1441 1.2 12.5 22.8 IMK+YT Sea water

Archaeplastida Rhodophyta

Bangiophyceae *Bangia*sp. AYCC145 0.8 39.0 0.5 IMK Sea water

Compsopogonophyceae *Erythlocladia*sp. AYCC144 5.8 17.9 6.9 IMK Sea water

*Erythropeltis* sp. AYCC180 4.5 13.6 3.8 IMK Sea water

*Erythrotrichia* sp. AYCC188 3.2 19.3 7.7 IMK Sea water

Unidentified AYCC192 0 19.9 0.1 IMK Sea water

Florideophyceae *Batrachospermumvirgato-decaisneanum* NIES-1458 61.8 30.9 25.6 C Freshwater

Porphyridiophyceae *Porphyridiumaerugineum* AYCC558 6.6 12.3 25.5 ESM** Terrestrial

*Porphyridiumsordidum* AYCC163 5.1 11.0 7.7 ESM*** BW

*Porphyridium* sp. AYCC36 4.8 5.3 5.8 IMK Sea water

*Porphyridium* sp. AYCC694 2.4 13.2 16.7 IMK Sea water

Rhodellophyceae *Corynoplastis japonica* AYCC232 2.0 12.3 11.1 IMK Sea water

*Dixoniellagrisea* OS 4 5.0 10.7 5.1 IMK Sea water

*Neorhodellacyanea* AYCC233 5.0 11.7 16.9 IMK Sea water

*Rhodella* sp. AYCC175 5.0 16.5 3.9 IMK Sea water

*Rhodella* sp. AYCC234 3.9 14.2 10.1 IMK Sea water

*Rhodella* sp. AYCC298 11.9 13.5 6.3 IMK Sea water

*Rhodellaviolacea* AYCC152 4.4 38.9 4.3 IMK Sea water

*Rhodellaviolacea* AYCC776 7.2 9.0 1.7 IMK Sea water

Stylonematophyceae *Bangiopsis*sp. AYCC550 5.2 39.6 2.5 IMK Sea water

*Chroodactylonornatum* AYCC82 0 13.3 10.7 IMK Sea water

*Rhodosorusmarinus* AYCC500 0 12.1 10.8 IMK Sea water

Charophyta

Charophyceae *Charabraunii* chara N 1.4 21.8 16.3 C Freshwater

Chlorokybophyta

Chlorokybophyceae *Chlorokybus* sp. NIES-160 8.3 21.2 19.7 AF6 Terrestrial

Chlorophyta

Chlorodendrophyceae *Tetraselmis* sp. nak 13 1.3 41.6 17.3 IMK Sea water

*Tetraselmis* sp. nak 14 0 20.4 10.9 IMK Sea water

*Tetraselmis*sp. nak 15 4.1 14.4 31.9 IMK Sea water

Chlorophyceae *Ankyra* sp. INB 86 6.7 15.2 18.5 AF6 Freshwater

*Asterococcussuperbus* NIES-1331 2.5 28.7 10.5 C Freshwater

*Carteria* sp. INB 95 3.5 30.5 20.8 AF6 Freshwater

*Chlamydomonas* sp. INB 83 13.1 31.0 7.4 AF6 Freshwater

*Chlamydomonasparkeae* NIES-440 14.8 17.6 8.9 IMK Sea water

*Chloromonas* sp. INB 36 8.6 27.5 0 AF6 Freshwater

*Coelastrum* sp. INB 97 13.8 31.2 9.7 AF6 Freshwater

*Desmodesmus* sp. INB 42 10.5 27.2 28.5 AF6 Freshwater

*Haematococcus* sp. nak 4 14.3 8.8 21.1 AF6 Freshwater

*Muriellazofingiensis* NIES-2175 12.3 12.3 30.3 C Terrestrial

*Mychonastes*sp. NIES-2341 9.2 20.0 23.1 C Freshwater

*Oedogoniumobesum* NIES-203 4.8 38.4 42.3 AF6 Freshwater

*Oedogonium*sp. nak 1001* 32.8 14.6 28.8 AF6 Freshwater

*Pandorina* sp. INB 25 12.3 14.2 0 AF6 Freshwater

*Paulschulziapseudovolvox* NIES-727 6.3 8.3 10.0 C Freshwater

*Pediastrum simplex* INB 88 6.2 25.2 23.9 AF6 Freshwater

*Phacotuslenticularis* NIES-859 4.2 20.0 14.9 C Freshwater

*Planktosphaeriagelatinosa* NIES-2268 8.7 27.6 19.1 C Freshwater

*Pleodorina*sp. INB 17 0.8 30.9 14.7 AF6 Freshwater

*Polyedriopsisspinulosa* INB 69 1.2 34.1 19.8 AF6 Freshwater

*Scenedesmus* sp. TIR 5 4.7 19.4 16.1 AF6 Freshwater

*Schroederiasetigera* NIES-246 11.2 28.1 15.1 C Freshwater

*Selenastrum* sp. INB 84 8.1 13.3 13.5 AF6 Freshwater

*Stigeocloniumaestivale* NIES-531 2.1 10.4 17.1 AF6 Freshwater

*Tetracystischlorococcoides* NIES-155 11.0 28.1 12.4 C Terrestrial

*Treubariatriappendiculata* INB 46 23.3 17.6 18.7 AF6 Freshwater

*Uronemaconfervicola* NIES-538 5.0 16.3 0 C Freshwater

Mamiellophyceae *Micromonaspusilla* NIES-1411 0 0 7.2 IMK Sea water

Nephroselmidophyceae *Nephroselmispyriformis* nak 11 17.2 15.9 11.5 IMK Sea water

Trebouxiophyceae *Auxenochlorellaprotothecoides* NIES-2176 10.4 25.6 13.2 AF6 Freshwater

*Chloroidiumellipsoideum* NIES-2150 13.4 16.6 30.3 C Freshwater

*Chloroidiumsaccharophilum* NIES-2352 14.5 23.0 5.8 AF6 Freshwater

*Choricystis*sp. NIES-1840 10.0 16.2 19.3 C Freshwater

*Dictyosphaerium*sp INB 72 17.2 14.3 22.2 AF6 Freshwater

*Micratctinium*sp. INB 16 13.6 14.4 11.8 AF6 Freshwater

*Oocystis*sp. INB 22 4.8 19.6 9.3 AF6 Freshwater

*Stichococcusbacillaris* NIES-529 31.2 3.9 29.0 C Freshwater

*Trebouxiaerici* NIES-2185 5.9 12.3 20.3 C Terrestrial

Ulvophyceae *Blidingia* sp. nak 22 5.9 16.2 8.8 IMK Sea water

*Halochlorococcum* sp. nak 21 30.5 1.5 15.8 IMK Sea water

*Oltmannsiellopsis* sp. nak 10 4.9 19.4 13.8 IMK Sea water

*Ulothrixvariabilis* NIES-329 58.2 20.4 11.8 C Freshwater

*Rhizochlonium* sp. nak 1002* 39.9 44.2 27.5 AF6 Freshwater

Unidentified nak 20 6.9 21.7 17.6 IMK Sea water

Incertaesedis *Pseudoscourfieldia marina* nak 16 10.1 19.1 20.4 IMK Sea water

*Pyramimoans*sp. nak 18 2.3 18.8 10.8 IMK Sea water

*Pyramimonas* sp. nak 19 16.0 26.0 30.1 IMK Sea water

Unidentified NIES-1435 8.0 14.4 15.6 IMK Sea water

Klebsormidiophyta

Klebsormidiophyceae *Klebsormidium*sp. nak 1 1.2 36.7 27.8 AF6 Terrestrial

Mesostigmatophyta

Mesostigmatophyceae *Mesostigmaviride* NIES-995 28.4 29.0 0 AF6 Freshwater

Tracheophyta

Magnoliopsida *Cabombacaroliana* We 1* 18.8 54.0 37.1 C Freshwater

*Egeriadensa* We 2* 20.6 32.6 56.9 C Freshwater

*Eleocharisacicularis* We 3* 32.4 42.1 34.9 C Freshwater

*Elodea nuttallii* We 4* 17.1 38.8 15.4 C Freshwater

*Lemnaaoukikusa* TIR 2* 51.1 33.5 14.4 C Freshwater

*Lemnaaoukikusa* TIR 3* 48.3 52.8 20.2 C Freshwater

Zygnematophyta

Zygnematophyceae *Closterium* sp. INB 91 12.5 15.5 20.7 AF6 Freshwater

*Desmidium*sp. INB 85 35.2 15.4 10.2 AF6 Freshwater

*Micrasterias*sp. nak 2 0 30.8 19.4 AF6 Freshwater

*Spirogyra* sp. nak 1003 67.5 25.9 23.5 AF6 Freshwater

Hacrobia Cryptophyta

Cryptophyceae *Chroomonas* sp. DA 28 0.7 13.5 14.1 IMK Sea water

*Hemiselmis* sp. DA 77 4.4 7.0 21.5 IMK Sea water

*Plagioselmis* sp. DA 34 1.9 10.4 7.1 IMK Sea water

*Rhodomonas*sp. DA 20 4.0 11.9 13.8 IMK Sea water

*Rhodomonas* sp. DA 41 8.0 19.0 5.7 IMK Sea water

Haptophyta

Coccolithophyceae *Calyptrosphaerasphaeroidea* nak 27 27.1 0 8.9 IMK Sea water

*Chrysochromulina* sp. nak 30 5.4 13.7 11.5 IMK Sea water

*Gephyrocapsaoceanica* nak 23 4.2 17.7 22.3 IMK Sea water

*Hymenomonas* sp. nak 25 0 19.7 17.8 IMK Sea water

*Imantonia* sp. nak 29 19.5 20.7 14.4 IMK Sea water

*Isochrysis*sp Is-ta-kw 5.5 14.7 2.0 IMK Sea water

*Phaeocystis* sp. nak 28 8.7 19.2 17.0 IMK Sea water

*Prymnesiumparvum* NIES-1017 13.0 38.2 9.6 IMK Sea water

Unidentified nak 26 0 10.7 5.9 IMK Sea water

Pavlovophyceae *Pavlova* sp. nak 33 1.0 24.4 13.7 IMK Sea water

*Pavlova*sp. nak 7 2.3 17.1 12.5 AF6 Freshwater

Rhizaria Cercozoa

Chrorarachniophyceae *Bigelowiellanatans* Kbn 1 4.6 6.9 8.2 IMK Sea water

Imbricatea *Ovulinataparva* NIES-2377 3.7 6.8 13.9 IMK+YT Sea water

Sarcomonadea *Cercomonaseffusa* NIES-2437 10.4 14.7 7.1 IMK+YT Sea water

Alveolata Dinophyta

Dinophyceae *Alexandriuminsuetum* NIES-678 1.6 11.5 11.4 IMK Sea water

*Amphidiniummassartii* TM 16 15.4 17.5 17.2 IMK Sea water

*Cooliamonotis* NIES-1833 0 19.3 5.4 IMK Sea water

*Heterocapsa* sp. DA 15 3.2 16.5 9.2 IMK Sea water

*Kareniamikimotoi* NIES-2411 1.8 14.1 14.8 IMK Sea water

*Prorocentrum* sp. DA 73 1.2 11.7 12.2 IMK Sea water

*Prorocentrumtriestnum* DA 74 0.5 25.0 5.4 IMK Sea water

*Thoracosphaeraheimii* NIES-1326 0 6.3 27.9 IMK Sea water

*Togulabritannica* NIES-405 2.9 17.5 24.2 IMK Sea water

Stramenopilea Bicosoecacea

Bicosoecea *Cafeteria roenbergensis* NIES-1012 6.0 6.2 0 IMK+YT Sea water

Pseudofungi

Bigyromonadea *Developayellaelegans* NIES-1388 2.4 2.4 30.1 IMK+YT Sea water

Ochrophyta

Aurearenophyceae *Aurearenacruciata* NIES-1865 9.0 14.2 11.3 IMK Sea water

Bacillariophyceae *Achnanthidiumminutissimum* NIES-71 5.8 5.8 14.4 CSi Freshwater

*Asterionellopsisglacialis* NIES-417 7.8 30.9 17.4 IMK Sea water

*Aulacoseiragranulata* NIES-333 13.0 16.3 0 CSi Freshwater

*Chaetocerosdebilis* Ch-deb-2 1.9 10.5 20.5 IMK Sea water

*Chaetocerosneogracile* Ch-n-kw 1.1 13.4 9.2 IMK Sea water

*Chaetocerossociale* NIES-553 16.5 29.4 21.2 IMK Sea water

*Chaetoceros*sp. ch-T3 1.5 15.1 15.6 IMK Sea water

*Chaetoceros*sp. ch-T1 2.5 21.8 8.6 IMK Sea water

*Cyclotellameneghiniana* NIES-805 8.4 6.7 10.6 AF6 Freshwater

*Cylindrothecaclosterium* NIES-1045 17.5 10.4 18.3 IMK Sea water

*Fragilariacapucina* NIES-391 31.2 31.2 12.7 AF6 Freshwater

*Hantzschiaamphioxys* sk-T2 3.0 14.8 14.7 CSi Freshwater

*Hantzschiaamphioxys* NIES-587 2.0 17.2 21.4 CSi Freshwater

*Neodenticulaseminae* Nd23×Nd36-3 0.0 21.1 11.5 IMK Sea water

*Nitzschiapalea* NIES-487 5.2 12.8 20.5 CSi Freshwater

*Skeletonema* sp. DA 123 25.4 34.3 7.4 AF6 Freshwater

*Thalassiosiranordenskioeldii* Th-n-5 0.0 15.0 19.1 IMK Sea water

Unidentified DA 109 10.8 21.5 15.3 IMK Sea water

Chrysomerophyceae *Giraudyopsis* sp. NIES-1862 4.8 14.4 25.5 IMK Sea water

Chrysophyceae *Epipyxisglabra* NIES-1826 6.2 38.9 15.5 AF6 Freshwater

*Paraphysomonasvestida* NIES-1377 9.5 12.2 7.1 AF6+YT Freshwater

*Poterioochromonasmalhamensis* NIES-2144 13.4 13.4 22.8 AF6 Freshwater

*Spumella* sp. NIES-1846 5.0 21.6 10.5 AF6+YT Freshwater

*Synuraspinosa* NIES-233 9.0 17.1 7.6 AF6 Freshwater

Dictyochophyceae *Rhizochromulina* sp. NIES-1382 7.0 19.9 17.7 AF6 Freshwater

Eustigmatophyceae *Nannochloropsisoculata* NIES-2146 6.3 18.3 14.9 IMK Sea water

Unidentified nak 9 55.0 34.8 28.4 AF6 Freshwater

*Vischeriapunctata* NIES-2147 0.6 22.4 26.5 C Freshwater

Pelagophyceae *Pelagomonascalceorata* NIES-1003 8.2 12.0 9.2 IMK Sea water

Phaeophyceae *Acinetosporacrinita* NIES-548 3.5 16.4 35.6 IMK Sea water

Raphidophyceae *Goniostomumdepressum* INB 123 3.4 17.6 10.5 AF6 Freshwater

*Heterosigmaakashiwo* DA 36 28.7 28.7 15.7 IMK Sea water

Schizocladiophyceae *Schizocladiaischiensis* NIES-1044 0 36.3 2.9 IMK Sea water

Xanthophyceae *Botrydiopsis* sp. TIR 1 0.8 25.7 3.7 AF6 Freshwater

*Ophiocytium* sp. nak 8 9.0 58.1 26.0 AF6 Freshwater

Incertaesedis *Olisthodiscusluteus* NIES-1831 0 32.5 21.4 IMK Sea water

Incertaesedis

Placidea *Wobblialunata* NIES-1015 0 3.7 9.3 IMK+YT Sea water

Incertaesedis Unidentified DA 96 0 5.4 3.3 IMK Sea water
